# Green-synthesized nanoparticles for glass ionomer cement reinforcement: a systematic review of their antimicrobial, mechanical, and physicochemical properties

**DOI:** 10.1186/s12903-026-09657-w

**Published:** 2026-10-03

**Authors:** Maged Mohamed, Hager Fawzy, Shahd Mahmoud, Mohamed Hani, Mohamed Abd El-Aal, Mohammad Asharf, Enas Elwakeel, Amr Azab, Dina Abozaid

**Affiliations:** 1 Faculty of Dental Medicine, Al-Azhar University, Cairo, Egypt; 2 Faculty of Dentistry, Tanta University, Tanta, Egypt; 3 Faculty of Dental Medicine, Fayoum University, Fayoum, Egypt; 4 Faculty of Dental Medicine, Al-Azhar University, Assuit, Egypt; 5 Chemistry Department, Faculty of Science, Assiut University, Assiut, 71516 Egypt; 6 Prosthodontics Department, Faculty of Oral and Dental Medicine, Alsalam University, Tanta, Egypt; 7 Dental Biomaterials Department, Faculty of Dentistry, Tanta University, Tanta, Egypt

**Keywords:** Glass ionomer cement, Green synthesized nanoparticles, Antimicrobial activity, Dental biomaterials, Nanoparticle incorporation, Biocompatibility

## Abstract

**Aims:**

This systematic review critically evaluates the antimicrobial performance, mechanical properties, and fluoride release of glass ionomer cements (GICs) modified exclusively with green-synthesized nanoparticles. The review highlights how eco-friendly nanoparticle production influences material behavior and clinical applicability.

**Methods:**

A comprehensive literature search was conducted across multiple electronic databases to identify in vitro, in vivo, and clinical studies reporting on GICs incorporated with green synthesized nanoparticles. Inclusion criteria were based on nanoparticle synthesis method, outcomes, and material characterization. Data extraction, quality assessment, and synthesis followed PRISMA guidelines.

**Results:**

Twenty-five studies met the inclusion criteria. Green-synthesized nanoparticles derived from plant and biological extracts consistently enhanced antibacterial activity through reactive oxygen species (ROS) generation, membrane disruption, ion release, and biofilm inhibition. Improvements in compressive strength, microhardness, and fluoride release were observed for several nanoparticle formulations, particularly chitosan–titania–zirconia–hydroxyapatite and plant-mediated hydroxyapatite. However, variability in synthesis routes, nanoparticle concentrations, and characterization protocols limited direct comparison across studies.

**Conclusions:**

Green-synthesized nanoparticles significantly improve the antimicrobial and mechanical properties of GICs while offering enhanced biocompatibility and sustainability compared to chemically synthesized nanomaterials. Standardized synthesis, precise nanoparticle characterization, and clinical validation are essential to advance the integration of green nanotechnology in restorative dentistry.

**Supplementary Information:**

The online version contains supplementary material available at https://doi.org/10.1186/s12903-026-09657-w.

## Introduction

Glass ionomer cements (GICs) remain indispensable restorative materials owing to their chemical adhesion to tooth structure, fluoride release, biocompatibility, and thermal compatibility with enamel and dentin. Nevertheless, conventional GICs exhibit relatively low mechanical strength and limited wear resistance, restricting their use in stress-bearing regions [1]. To overcome these shortcomings, various nanoparticles have been incorporated into GIC formulations to improve antibacterial activity, mechanical performance, and overall clinical durability while preserving their inherent biological advantages [2].

Numerous studies have investigated the addition of various chemically synthesized nanoparticles, such as silver, gold, titanium dioxide, and hydroxyapatite, into GICs [2]. These nanoparticles have shown promise in improving compressive strength, antimicrobial efficacy, and filling of micro-voids in the cement matrix. However, these conventional nanoparticle-modified GICs often face challenges including poor dispersion leading to agglomeration, inconsistent mechanical performance under different conditions, and concerns about cytotoxicity and long-term biocompatibility [2]. Some studies reported insignificant effects on mechanical properties or issues with nanoparticle-induced color changes, limiting clinical acceptance.

Green synthesized nanoparticles have emerged as a promising solution to these drawbacks. Produced using biological agents such as plant extracts (e.g., miswak, ginger, clove, and *Zingiber* spp.), green nanoparticles offer safer, eco-friendly synthesis with minimal toxic by-products. They exhibit enhanced biocompatibility and reduced cytotoxicity compared to chemically synthesized counterparts, addressing safety concerns in clinical dental applications [1]. Additionally, green nanoparticles benefit from bioactive compounds naturally present in their synthesis agents, which often confer superior antimicrobial properties, improving infection control and reducing postoperative sensitivity. These characteristics have prompted increasing interest in the incorporation of green-synthesized nanoparticles into glass ionomer cements for restorative dental applications.

Green synthesized nanoparticles are formed through an eco-friendly and sustainable process that utilizes natural biological agents such as plant extracts, microorganisms, or bio-compounds to reduce metal ions into nanoparticles [1, 3]. Plant extracts contain a rich mixture of phytochemicals, including phenolics, flavonoids, terpenoids, alkaloids, sugars, and organic acids, that act as both reducing and stabilizing agents [4]. During synthesis, metal ions from precursors (e.g., silver nitrate) interact with these biomolecules, which donate electrons to reduce the metal ions into their zero-valent nanoparticle forms. Subsequently, other biomolecules function as capping agents that stabilize the particle surface, preventing aggregation and controlling particle size and shape [3, 4].

The mechanism of antimicrobial action of green synthesized nanoparticles primarily involves the generation of reactive oxygen species (ROS) such as hydroxyl radicals and hydrogen peroxide, which cause oxidative stress, damaging bacterial cell membranes and intracellular components [3, 5]. Additionally, nanoparticles can penetrate the bacterial cell walls, disrupting membrane integrity and interfering with vital processes such as respiration, DNA replication, and enzyme activity. The natural bioactive compounds adsorbed on green nanoparticles may also enhance antibacterial effects by synergistically interacting with microbial cells. This dual physical and biochemical mode of action results in superior antimicrobial activity, making green synthesized nanoparticles especially effective for incorporation into glass ionomer cements to improve their resistance against oral pathogens while maintaining biocompatibility [6].

Although several recent investigations have explored the incorporation of nanoparticles into glass ionomer cements, these studies have primarily focused on individual nanoparticle formulations or included chemically synthesized nanoparticles alongside green-synthesized materials. To the best of our knowledge, no previous systematic review has comprehensively synthesized the evidence regarding exclusively green-synthesized nanoparticles incorporated into GICs while simultaneously evaluating their antimicrobial performance, mechanical properties, fluoride release, color stability, and other physicochemical characteristics. Given the increasing emphasis on sustainable biomaterials and eco-friendly nanotechnology in restorative dentistry, a focused synthesis of this emerging evidence is warranted. Therefore, the present systematic review aimed to critically evaluate the available evidence regarding green-synthesized nanoparticles incorporated into GICs and their effects on antimicrobial activity, mechanical performance, fluoride release, and related material properties.

Accordingly, this systematic review addressed the following focused research question: Does the incorporation of green-synthesized nanoparticles into glass ionomer cements alter their antibacterial activity, mechanical properties, physicochemical properties, fluoride release, and color stability compared with conventional glass ionomer cements?

## Methodology

### Study design & search strategy

This systematic review was conducted and reported in accordance with the Preferred Reporting Items for Systematic Reviews and Meta-Analyses (PRISMA) guidelines [7] and the Cochrane Handbook for Systematic Reviews of Interventions [8]. The review protocol was prospectively registered in the PROSPERO database (Registration No. CRD420251231831).

#### Eligibility criteria

The eligibility criteria were established using the Population, Intervention, Comparator, Outcomes (PICO) framework. The population comprised glass ionomer cement (GIC), the intervention was the incorporation of green-synthesized nanoparticles, the comparator was conventional (unmodified) GIC, and the outcomes included antibacterial activity, mechanical properties, fluoride release, color stability, and other physicochemical properties. Based on the PICO framework, the review addressed the following focused research question: Does the incorporation of green-synthesized nanoparticles into glass ionomer cements alter their antibacterial activity, mechanical properties, physicochemical properties, fluoride release, and color stability compared with conventional glass ionomer cements?

##### Inclusion criteria

Studies were included if they:

- Were in vitro, in vivo animal, or clinical studies.
- Evaluated glass ionomer cement modified with green-synthesized nanoparticles.
- Included conventional (unmodified) glass ionomer cement as the comparator.
- Reported at least one predefined outcome, including antibacterial activity, mechanical properties, fluoride release, color stability, or other physicochemical properties.
- Provided quantitative outcome data sufficient for data extraction and synthesis.

##### Exclusion criteria

Studies were excluded if they:

- Were reviews, systematic reviews, meta-analyses, case reports, conference abstracts, editorials, letters, or book chapters.
- Evaluated chemically synthesized nanoparticles or nanoparticles produced by methods other than green synthesis.
- Investigated restorative materials other than glass ionomer cement.
- Did not report any of the predefined outcome measures.
- Did not provide sufficient quantitative data for extraction or analysis.

Electronic database searches were conducted in PubMed, Web of Science, Scopus, and the Cochrane Library from database inception to January 2025. The search strategy combined controlled vocabulary and free-text terms related to "glass ionomer cement," "nanoparticles," and "green nanotechnology" using the Boolean operators AND, OR, and NOT. The complete search strategy for each database is presented in Table 1. No restrictions were applied regarding language or publication date.

**Table 1:** Presents full search strategy for electronic databases

Database	Search strategy
Pubmed	(Nanoparticles OR "Nano particles" OR Nanomaterials OR "Nanoscale particles") AND ("Glass ionomer cement" OR GIC OR "Glass polyalkenoate cement" OR "polyalkenoate cement")
Scopus	
Web of science	
Cochrane	

Following duplicate removal, two reviewers independently screened titles and abstracts for eligibility (M.M. and A.A. or M.E. and M.A.), followed by full-text assessment. Any disagreements were resolved through discussion, with consultation of a third reviewer (D.A.) when necessary. Only studies fulfilling all predefined eligibility criteria were included in the final review.

All retrieved records were imported into Rayyan [9], where duplicate records were identified and removed. The remaining studies were divided into two groups and independently screened by two reviewers using the predefined eligibility criteria. Full-text articles were subsequently evaluated to confirm study eligibility. Inter-reviewer agreement was assessed using Jamovi statistical software [10].

Data extraction was performed independently by four reviewers (A.A., M.A., M.M., and M.E.) using a standardized data extraction form. The extracted information included study characteristics (sample size, GIC brand, source of the green synthesis agent, extraction method, and experimental groups), nanoparticle characteristics, and outcome data related to antibacterial activity, mechanical properties, fluoride release, color stability, and other physicochemical properties.

A quantitative meta-analysis was not performed because of substantial methodological and clinical heterogeneity among the included studies, including differences in nanoparticle composition, synthesis methods, concentrations, glass ionomer cement formulations, outcome measures, and testing protocols. Therefore, a qualitative systematic synthesis of the evidence was undertaken.

### Quality assessment method

The risk of bias in included in vitro studies was assessed independently by at least by two reviewers (M.M and A.A) or (M.E and M.A) using study design-specific risk-of-bias tools. Disagreements between reviewers were resolved through discussion until consensus was achieved. Reviewers assessed inter-rater agreement using Jamovi statistics software [10].

The Quality Assessment Tool for In Vitro Studies (QUIN) was used to evaluate the methodological quality of all included in vitro studies [11]. The QUIN tool comprises 12 methodological criteria assessing the clarity of study objectives, sample size calculation, sampling technique, comparison groups, methodological description, operator details, randomization, blinding, outcome assessment, outcome reporting, statistical analysis, and presentation of results. Each criterion was scored as adequately specified (2 points), inadequately specified (1 point), or not specified (0 points). The overall quality score was calculated as the percentage of the maximum applicable score using the following formula:

$$\mathrm{QUIN}\;\mathrm{Score}\;(\%)=\frac{\mathrm{Total}\;\mathrm{score}\;\mathrm{obtained}}{2\times\mathrm{Number}\;\mathrm{of}\;\mathrm{applicable}\;\mathrm{criteria}}\times100$$

Studies were categorized according to the original QUIN classification as low risk of bias (> 70%), medium risk of bias (51–70%), or high risk of bias (≤ 50%).

Because one included study involved an in vivo animal experiment, its methodological quality was assessed separately using the SYRCLE Risk of Bias tool (Systematic Review Centre for Laboratory Animal Experimentation) [12].

## Results

### Study characteristics

**Fig. 1:**
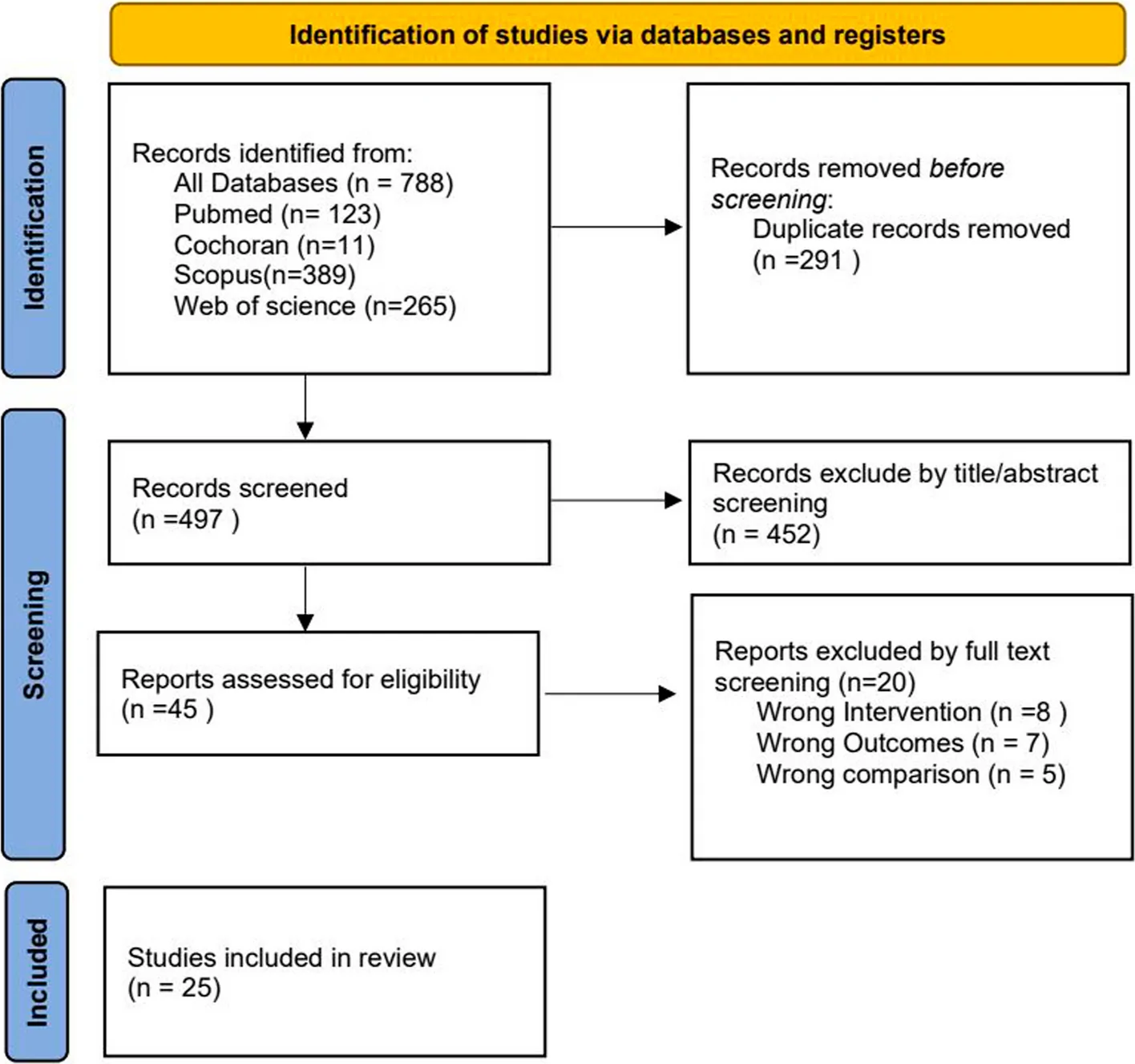
PRISMA flow chart of included studies

The initial database search identified 788 records, of which 291 duplicates were removed, leaving 497 unique articles for title and abstract screening. Following preliminary assessment, 471 studies were excluded for failing to meet the inclusion criteria, and 26 articles underwent full-text evaluation. Ultimately, 25 studies satisfied all selection requirements and were included in the final synthesis, comprising 24 in vitro studies and one in vivo animal study. The PRISMA flow diagram summarizing the study selection process is presented in Fig. 1, while the characteristics of the included studies are summarized in Table 2.

**Table 2:** Presents summary of included studies

Significance/Key Findings	Related Measured Outcomes	Aim/Purpose of the Study	Study
Incorporation of ZnO nanoparticles significantly improved antibacterial activity against *S. mutans*; ZnO-GIC may prevent recurrent caries	Antibacterial activity	To evaluate antibacterial activity of ZnO nanoparticle-incorporated GIC	Ahalya et al. 2021 [13]
10% Ch-Ti-Zr-HA modified GIC enhanced compressive and flexural strength; improved hardness; suitable for high-stress restorations	Cytotoxicity, inflammation, antimicrobial activity, mechanical properties, solubility	To evaluate biocompatibility, antimicrobial, anti-inflammatory, and mechanical properties of phyto-mediated nanocomposite-modified GIC	Alex et al. 2025 [14]
AgNPs, miswak extract, CHX, and combined AgNPs + CHX can enhance antimicrobial activity; potential to enhance caries prevention	Inhibition zones, compressive strength	To compare antimicrobial efficacy and compressive strength of GIC modified with biosynthesized AgNPs, miswak extract, CHX, and combinations	Ashour et al. 2022 [15]
Enhanced antimicrobial properties reduce secondary caries risk; copper and silver NPs improve antibacterial activity	Inhibition zones, MDCT colony counts, compressive strength, drug release	To evaluate antimicrobial efficacy and compressive strength of GIC modified with CuNPs, AgNPs, and/or metronidazole	Ashour et al. 2022 [16]
Nanoparticles influence fluoride release profile in GIC	Fluoride release	To compare fluoride release between nano-formulated and conventional GICs	Dhivyasri et al. 2025 [17]
Green-synthesized AgNPs improved antibacterial activity without harming mechanical properties	Antibacterial activity, microhardness, color change (before/after thermocycling)	To assess antibacterial activity, microhardness, and color stability of GIC modified with green AgNPs and Ag@MoS₂ NCs	Ekrikaya et al. 2025 [18]
AgNPs + amoxicillin showed synergistic antimicrobial effect with minimal impact on compressive strength	Antimicrobial activity, compressive strength	To synthesize AgNPs and incorporate them with amoxicillin into GIC to enhance antimicrobial activity	Enan et al. 2021 [19]
Gold and zirconia nanoparticles showed strong inhibition of *S. mutans* and *L. acidophilus*; effective for orthodontic applications	Antimicrobial activity	To evaluate antimicrobial effects of orthodontic band cement incorporated with biosynthesized nanoparticles	Faizee et al. 2024 [20]
Plant-based nanoparticles (Ti, Zr, HA) improved wettability and reduced surface roughness of GIC	Surface roughness, wettability, surface morphology	To evaluate how plant-based nanoparticles affect wettability and surface roughness of GIC	Ganesh et al. 2024 [21]
AgNW-modified GIC showed strong antibiofilm activity with preserved mechanical/biochemical properties	Antibiofilm efficacy; mechanical/biochemical properties	To evaluate antibiofilm effect and properties of AgNW-modified GIC	Guo et al. 2025 [22]
Green-synthesized nHA improved antibacterial activity, compressive strength, fluoride release, and biocompatibility	Compressive strength, fluoride release, antibacterial effect	To evaluate antimicrobial properties and compressive strength of nHA-incorporated GIC	Ilancheran et al. 2024 [23]
Green-synthesized ZrO₂ nanoparticles improved strength and antibacterial effects	Compressive strength; antibacterial activity	To assess antimicrobial efficacy and compressive resilience of ZrO₂-modified GIC	Jain et al. 2024 [24]
Improved mechanical and antibacterial properties, with clinical benefits particularly for pediatric dentistry	Mechanical properties	To enhance mechanical performance of GIC using green-mediated modifications	Jairam et al. 2024 [25]
TiO₂ NPs synthesized via *Bacillus subtilis* improved mechanical properties and biocompatibility of GIC	Mechanical properties, biocompatibility	To develop TiO₂ nanoparticle-modified GIC for caries treatment	Mansoor et al. 2022 [26]
Titania-enriched GIC improved enamel bond strength and extended storage life; eco-friendly enhancement	Mechanical properties, enamel shear bond strength	To synthesize TiO₂ from corn silk fibers and incorporate into GIC to create a smart/eco-friendly restorative material	Mansoor et al. 2025 [27]
Nano-HAp incorporation affected water sorption and solubility of GIC and resin cements	Water sorption, solubility	To evaluate water sorption and solubility of nano-HAp modified GIC and resin luting cements	Mariappan et al. 2025 [28]
Green-synthesized AgNPs from mint extract enhanced antibacterial activity of GIC	Antibacterial activity	To evaluate antibacterial activity of mint-mediated AgNP-modified GIC	Goldani Moghadam et al. 2022 [29]
Nanoparticle-modified GIC improved color stability and reduced surface abrasion	Color stability; surface abrasion	To evaluate color stability and abrasion resistance of nanoparticle-modified GIC with dentifrices	Palani et al. 2025 [30]
Biosynthesized SNPs improved microhardness, compressive strength, and color stability; reduced porosity	Microhardness, compressive strength, color stability, nano-CT porosity	To evaluate mechanical and optical properties and internal porosity of SNP-modified GIC	Qasim et al. 2021 [31]
Ch-Ti-Zr-HA modified GIC showed improved mechanical properties after thermocycling	Compressive strength, flexural strength, microhardness	To assess thermocycling effects on green-mediated nanocomposite-modified GIC	Ravi et al. 2024 [32]
SI-AgNPs enhanced antibacterial properties of GIC; sustainable and eco-friendly	Antibacterial activity	To evaluate antibacterial activity of SI-AgNPs alone and incorporated into GIC	Siddiqui et al. 2024 [33]
Green-mediated nanocomposite (Ch-Ti-Zr-HA) improved wear resistance of GIC	Wear resistance	To investigate wear resistance of nanocomposite-modified GIC	Sitaram et al. 2024 [34]
Mango leaf-mediated AgNPs improved GIC mechanical and antibacterial properties	Antibacterial activity	To synthesize green AgNPs using mango leaves and evaluate their effect on GIC	Sundeep et al. 2017 [35]
Multifaceted approach to combat dental caries, lesions, and microbial resistance; Enhances patient outcomes in dentistry	Antibacterial activity Surface energetics	To investigate the green synthesis of cephalosporin-capped copper nanoparticles (CuNPs) from Mentha longifolia extract and evaluate their antibiofilm properties through the extended Derjaguin–Landau–Verwey–Overbeek (XDLVO) approach when supplemented with glass ionomer cement (GIC)	Saddique et al. 2025 [36]

The included studies represented a broad geographical distribution, with the majority originating from India (12 studies), followed by Pakistan (three studies), Saudi Arabia (three studies), and individual contributions from China, Kuwait, and Turkey. The studies collectively investigated a wide range of green-synthesized nanoparticles, including silver, copper, zirconia, hydroxyapatite, gold, chitosan-based hybrids, and multi-component systems such as chitosan–titania–zirconia–hydroxyapatite. Nanoparticle concentrations varied substantially across studies, ranging from 0.05% to 10% by weight, and were incorporated into either the powder, liquid, or both phases of the GIC formulation (Table 3).

**Table 3:** Presents study characteristics

Study	Country	Sample Size	Nanoparticle Source	Nanoparticle Type	NP Size	Characterization Techniques	GIC Brand
Palani et al. 2025 [30]	India	8	Chitosan	Chitosan–TiO₂–ZrO₂–HA	—	FESEM (Jeol IT800-SHL, 7 kV)	GC Corporation, Tokyo
Qasim et al. 2021 [31]	Kuwait	5 for hardness; 6 for CS; 10 for color	Chitosan	Silver NPs	122 nm	ICP-OES; TEM; FTIR-ATR (Bruker Tensor 27)	Ketac CEM (3M GmbH, Germany)
Ravi et al. 2024 [32]	India	12	Chitosan, Neem, Moringa	Ch-Ti-Zr-HA NPs	—	Not reported	—
Siddiqui et al. 2024 [33]	Pakistan	—	*Salsola imbricata*	Silver NPs	20–50 nm (SEM)	UV–Vis; SEM–EDX; FTIR	GC Corporation (Japan)
Saddique et al. 2025 [36]	Pakistan	96-well plates	Mentha longifolia leaf extract	copper nanoparticles	Below 50 nm	Chemical characterization UV–Vis spectroscopy Fourier transform infrared spectroscopy FEG-SEM	GIC® (Shanghai New Century Dental Materials Co., Ltd.)
Sitaram et al. 2024 [34]	India	10	Eucalyptus-based chitosan; Neem-based TiO₂	Ch-Ti-Zr-HA NPs	—	Not reported	—
Sundeep et al. 2017 [35]	India	5	*Mangifera indica*	Silver NPs	31.7 nm (DLS)	DLS; PXRD; SEM; EDX; UV–Vis	—
Dhivyasri et al. 2025 [17]	India	—	Eucalyptus, Aloe vera, Moringa, Neem	Ch-Ti-Zr-HA NPs	—	—	Nano-formulated GIC
Faizee et al. 2024 [20]	Saudi Arabia	—	White tea, Dry ginger	ZrO₂, Au, Cu NPs	—	SEM; TEM; FTIR	GC Fuji GIC
Alex et al. 2025 [14]	India	0.6 effect size	Eucalyptus, Neem, Aloe vera, Moringa	Nano-composite Ch-Ti-Zr-HA	—	UV–Vis; XRD; SEM; FTIR	Type IX GIC (GC Corporation)
Ekrikaya et al. 2025 [18]	Turkey	10 mm × 2 mm discs	Green tea extract	Silver NPs & Ag@MoS₂ NC	—	FTIR; UV–Vis; SEM–EDX; DLS	GC Fuji IX
Ashour et al.2022 [15]	Saudi Arabia	—	*Zingiber officinale*, *Salvadora persica*	ZOE-AgNPs	10.5–14.12 nm	UV–Vis; TEM; XRD; FTIR	GC Fuji IX
Ashour et al. 2022 [16]	Saudi Arabia/Egypt	180 discs; CS: 8/group	*Thymus vulgaris*	CuNPs, AgNPs	20–50 nm	UV–Vis; TEM; XRD; FTIR	GC Fuji IX
Ahalya et al. 2021 [13]	India	—	Clove	ZnO NP	86 nm	UV–Vis; FTIR; XRD; SEM	—
Enan et al. 2021 [19]	Saudi Arabia/Egypt	—	*Cupressus macrocarpa*	Silver biogenic NPs	—	UV–Vis; TEM; XRD; FTIR	Fuji IX GP
Ganesh et al. 2024 [21]	India	—	Eucalyptus, Aloe vera, Moringa, Neem	TiO₂, ZrO₂, HA, Chitosan NPs	—	AFM; SEM; Contact angle	GC Corporation
Guo et al. 2025 [22]	China	—	—	Ag nanowires & AgNPs	AgNW 120 nm × 45 µm; AgNP 60–120 nm	SEM; EPS assay; Live/dead assay; Lactic acid assay	—
Ilancheran et al. 2024 [23]	India	Effect size 0.6	*Moringa oleifera*	Nano-hydroxyapatite	—	FTIR; SEM; EDX; MIC; ELISA	Fuji IX GP/Fuji II
Jain et al. 2024 [24]	India	Effect size 0.6	*Aloe vera*	Zirconia NPs	—	FTIR; SEM; EDX; Antibacterial test	GC Corporation
Jairam et al. 2024 [25]	India	—	Mushrooms	Cerium oxide NPs	~ 5 nm	XRD; FTIR; SEM; EDAX; HRTEM	Fuji II (GC America)
Mansoor et al. 2022 [26]	Pakistan	—	*Bacillus subtilis*	TiO₂ NPs	~ 70 nm	XRD; SEM; TEM; FTIR; AFM; DRS; EDX	Rigaku
Mansoor et al. 2025 [27]	Pakistan	—	Green synthesis	TiO₂ NPs	24 nm (XRD); 35 nm (DLS)	XRD; SEM; FTIR; DLS	GC Universal Restorative Cement 2
Mariappan et al. 2025 [28]	India	80	Eggshell-derived hydroxyapatite	Nano-HAp	100–200 nm	XRD; SEM–EDAX; FTIR	GC Gold Label 1
Goldani Moghadam et al. 2022 [29]	Iran	90 (30/group)	*Mentha piperita*	Silver NPs	71.67 nm	TEM; DLS; Disc diffusion	Fuji II LC

### Quality assessment results

Among the included studies, 24 were in vitro investigations and one was an in vivo animal study. Accordingly, the in vitro studies were assessed using the QUIN tool, whereas the animal study was evaluated using the SYRCLE Risk of Bias tool. The methodological quality of the included in vitro studies was generally moderate to high, with 11 studies classified as low risk of bias and 13 studies as medium risk of bias according to the QUIN tool.

Of the 24 studies, 11 studies (45.8%) were classified as having a low risk of bias, while 13 studies (54.2%) were classified as having a medium risk of bias. No study was classified as having a high risk of bias according to the QUIN classification. Detailed quality assessment results for the in vitro studies are presented in Table 4.

**Table 4:** Quality assessment results of included studies

Study	Clearly stated aims and objectives	Sample-size calculation	Detailed explanation of the sampling technique	Details of the comparison group	Detailed explanation of the methodology	Operator details	Randomization	Blinding	Measurement of outcomes	Outcome-reporting details	Statistical analysis	Presentation of results	Total score out of 24	Percentage	Risk of bias
Ahalya et al., 2021 [13]	2	0	1	2	2	0	0	0	2	2	0	2	13	54.2%	Medium
Alex et al., 2025 [14]	2	2	1	2	2	0	0	0	2	2	2	2	17	70.8%	Low
Ashour et al., 2022 [15]	2	0	1	2	2	0	0	0	2	2	2	2	15	62.5%	Medium
Ashour et al., 2022 [16]	2	0	1	2	2	0	0	0	2	2	2	2	15	62.5%	Medium
Dhivyasri et al., 2025 [17]	2	2	1	2	2	0	0	0	2	2	2	2	17	70.8%	Low
Ekrikaya et al., 2025 [18]	2	2	1	2	2	0	0	0	2	2	2	2	17	70.8%	Low
Enan et al., 2021 [19]	2	0	1	2	2	0	0	0	2	2	2	2	15	62.5%	Medium
Faizee et al., 2024 [20]	2	0	1	2	1	0	0	0	2	1	2	2	13	54.2%	Medium
Ganesh et al. 2024 [21]	2	2	1	2	2	0	0	0	2	2	2	2	17	70.8%	Low
Guo et al., 2025 [22]	2	0	1	2	2	0	0	0	2	2	2	2	15	62.5%	Medium
Ilancheran et al., 2024 [23]	2	2	1	2	2	0	0	0	2	2	2	2	17	70.8%	Low
Jain et al., 2024 [24]	2	2	1	2	2	0	0	0	2	2	2	2	17	70.8%	Low
Jairam et al., 2024 [25]	2	0	1	2	2	0	0	0	2	2	2	2	15	62.5%	Medium
Mansoor et al., 2022 [26]	2	0	1	2	2	0	0	0	2	2	1	2	14	58.3%	Medium
Mansoor et al., 2025 [27]	2	0	1	2	2	0	0	0	2	2	2	2	15	62.5%	Medium
Mariappan et al., 2025 [28]	2	2	1	2	2	0	0	0	2	2	2	2	17	70.8%	Low
Goldani Moghadam et al. 2022 [29]	2	0	1	2	2	0	0	0	2	2	2	2	15	62.5%	Medium
Palani et al. 2025a [30]	2	2	2	2	2	2	2	2	2	2	2	2	24	100.0%	Low
Qasim et al., 2021 [31]	2	2	1	2	2	2	0	2	2	2	2	2	21	87.5%	Low
Ravi et al., 2024 [32]	2	2	1	2	2	0	0	0	2	2	2	2	17	70.8%	Low
Saddique et al., 2025 [36]	2	0	1	2	2	0	0	0	2	2	1	2	14	58.3%	Medium
Siddiqui et al., 2024 [33]	2	0	1	2	2	0	0	0	2	2	2	2	15	62.5%	Medium
Sitaram et al., 2024 [34]	2	2	1	2	2	0	0	0	2	2	2	2	17	70.8%	Low
Sundeep et al., 2017 [35]	2	0	1	2	2	0	0	0	2	2	0	2	13	54.2%	Medium

Scoring: 2 = adequately specified; 1 = inadequately specified; 0 = not specified. The maximum possible score is 24

One included study investigated the biocompatibility of nano-modified glass ionomer cement using a Wistar rat model and was therefore assessed using the SYRCLE Risk of Bias tool [37]. The study demonstrated a moderate overall risk of bias. The detailed domain-specific assessment is provided in Supplementary Table S3.

### Antibacterial activity results

Antibacterial activity was evaluated in 16 of the 25 included studies (64%), making it the most frequently investigated outcome. Across the included studies, green-synthesized nanoparticles consistently enhanced antibacterial activity against common oral pathogens, irrespective of nanoparticle composition, although the magnitude of improvement varied between formulations.

Across all included studies, plant-mediated silver nanoparticles produced the most consistent antibacterial enhancement, whereas hydroxyapatite-, zirconia-, and chitosan-based nanoparticles also demonstrated favorable antibacterial performance, although the magnitude of improvement varied according to nanoparticle composition and concentration (Table 5).

**Table 5:** Antimicrobial activity results

Study	Groups	Bacteria	Test	Time point	Mean (SD)	
Alex et al. 2025 [14]	GIC + 3% Ch-Ti-Zr-HA Nps	*S. mutans*	Time kill curve assay	1 h	408.47	
				2 h	409.6	
				3 h	426.55	
				4 h	385.88	
				5 h	322.6	
	GIC + 5% Ch-Ti-Zr-HA Nps			1 h	403.95	
				2 h	406.21	
				3 h	418.64	
				4 h	385.88	
				5 h	320.34	
	GIC + 10% Ch-Ti-Zr-HA Nps			1 h	401.69	
				2 h	401.69	
				3 h	423.16	
				4 h	357.63	
				5 h	319.21	
	GIC			1 h	559.89	
				2 h	579.1	
				3 h	594.92	
				4 h	617.51	
				5 h	651.41	
	GIC + 3% Ch-Ti-Zr-HA Nps	*Lactobacillus*		1 h	469.12	
				2 h	461.76	
				3 h	413.24	
				4 h	380.88	
				5 h	351.47	
	GIC + 5% Ch-Ti-Zr-HA Nps			1 h	423.53	
				2 h	351.47	
				3 h	319.12	
				4 h	252.94	
				5 h	207.35	
	GIC + 10% Ch-Ti-Zr-HA Nps			1 h	410.29	
				2 h	358.82	
				3 h	313.24	
				4 h	267.65	
				5 h	207.35	
	GIC			1 h	525	
				2 h	558.82	
				3 h	686.76	
				4 h	614.71	
				5 h	657.35	
Faizee et al. 2024 [20]	ZrNps	*S mutans* and *L acidophilus*	Time Kill Curve Assay	1 h	Between groups (Mean Square)	0.001
	AuNps				within groups	0.003
	CuNps			2 h	Between groups	0.001
					within groups	0.006
	control			3 h	Between groups	0.001
					within groups	0.011
				4 h	Between groups	0.001
					within groups	0.017
Ilancheran et al. 2024 [23]	3% nHA incorporated GIC	*S. mutans*	minimal inhibitory concentration (MIC)	1 h	0.567 ± 0.00083	
				2 h	0.522 ± 0.00051	
				3 h	0.498 ± 0.00081	
				4 h	0.452 ± 0.00121	
				5 h	0.420 ± 0.00051	
	5% nHA incorporated GIC			1 h	0.521 ± 0.00051	
				2 h	0.486 ± 0.00051	
				3 h	0.454 ± 0.00063	
				4 h	0.411 ± 0.00051	
				5 h	0.365 ± 0.00051	
	10% nHA incorporated GIC			1 h	0.487 ± 0.00103	
				2 h	0.464 ± 0.00075	
				3 h	0.421 ± 0.00054	
				4 h	0.365 ± 0.00103	
				5 h	0.314 ± 0.00083	
	control			1 h	0.603 ± 0.00126	
				2 h	0.631 ± 0.00051	
				3 h	0.672 ± 0.0008	
				4 h	0.698 ± 0.00132	
				5 h	0.631 ± 0.00051	
	3% nHA incorporated GIC	*Lactobacillus*		1 h	0.764 ± 0.00051	
				2 h	0.731 ± 0.00054	
				3 h	0.694 ± 0.00051	
				4 h	0.662 ± 0.00051	
				5 h	0.621 ± 0.00081	
	5% nHA incorporated GIC			1 h	0.733 ± 0.00054	
				2 h	0.701 ± 0.00051	
				3 h	0.671 ± 0.00051	
				4 h	0.622 ± 0.00051	
				5 h	0.588 ± 0.00103	
	10% nHA incorporated GIC			1 h	0.711 ± 0.00121	
				2 h	0.652 ± 0.00051	
				3 h	0.611 ± 0.00054	
				4 h	0.565 ± 0.00089	
				5 h	0.511 ± 0.00054	
	control			1 h	0.831 ± 0.00051	
				2 h	0.833 ± 0.00054	
				3 h	0.866 ± 0.00051	
				4 h	0.897 ± 0.00213	
				5 h	0.822 ± 0.00081	
Ekrikaya et al. 2025 [18]	GIC (control group)	*S. mutans*	MTT activity	24-h	0.587 (0.587)	
	GIC + Green synthesized Ag NPs				0.315 (0.369)	
	GIC + Green synthesized Ag@MoS2 nanocomposite				0.258 (0.214)	
	GIC + Chemically synthesized Ag NPs				0.298 (0.758)	
	GIC + Chemically synthesized Ag@MoS2				0.214 (0.321)	
	GIC (control group)	*S. mutans*	Live Bacteria (%)	18–24-h-old	78.32 (0.541)	
	GIC + Green synthesized Ag NPs				38.23 (0.325)	
	GIC + Green synthesized Ag@MoS_2_ nanocomposite				31.25 (0.145)	
	GIC + Chemically synthesized Ag NPs				39.78 (0.236)	
	GIC + Chemically synthesized Ag@MoS_2_				34.36 (0.785)	
	GIC (control group)	*S. mutans*	Agar disk diffusion test	24 h	7.054 (0.123)	
	GIC + Green synthesized Ag NPs				18.08 (0.271)	
	GIC + Green synthesized Ag@MoS_2_ nanocomposite				21.07 (0.113)	
	GIC + Chemically synthesized Ag NPs				16.147 (0.178)	
	GIC + Chemically synthesized Ag@MoS_2_				20.123 (0.352)	
	GIC (control group)		Lactic acid production		11.425 (0.145)	
	GIC + Green synthesized Ag NPs				9.153 (0.054)	
	GIC + Green synthesized Ag@MoS_2_ nanocomposite				6.14 (0.078)	
	GIC + Chemically synthesized Ag NPs				9.939 (0.178)	
	GIC + Chemically synthesized Ag@MoS_2_				7.874 (0.178)	
Ashour et al. 2022 [15]	GIC + Lyophilized miswak	*Staphylococcus aureus*	Disc diffusion test	24 h	18.1 (1.9)	
	GIC + ZOE-AgNPs				19.4 (2.1)	
	GIC + Chlorhexidine diacetate (CHX)				20.7 (1.5)	
	GIC + ZOE-AgNPs + CHX				26.2 (2.6)	
	Control (Conventional GIC)				9.2 (1.2)	
	GIC + Lyophilized miswak			1 week	12.2 (1.2)	
	GIC + ZOE-AgNPs				14.4 (1.1)	
	GIC + Chlorhexidine diacetate (CHX)				16.4 (1.6)	
	GIC + ZOE-AgNPs + CHX				22.2 (2.1)	
	Control (Conventional GIC)				0 (0)	
	GIC + Lyophilized miswak			3 weeks	9.3 (1.1)	
	GIC + ZOE-AgNPs				12.1 (0.9)	
	GIC + Chlorhexidine diacetate (CHX)				14.4 (1.5)	
	GIC + ZOE-AgNPs + CHX				19.1 (1.7)	
	Control (Conventional GIC)				0 (0)	
	GIC + Lyophilized miswak	*S. mutans*		24 h	17.98 (1.31)	
	GIC + ZOE-AgNPs				18.93(1.67)	
	GIC + Chlorhexidine diacetate (CHX)				20.12(1.78)	
	GIC + ZOE-AgNPs + CHX				26.19(0.95)	
	Control (Conventional GIC)				9.17(1.43)	
	GIC + Lyophilized miswak			1 week	12.02(1.31)	
	GIC + ZOE-AgNPs				14.29(1.07)	
	GIC + Chlorhexidine diacetate (CHX)				16.19(0.95)	
	GIC + ZOE-AgNPs + CHX				22.14(1.07)	
	Control (Conventional GIC)				0(1.31)	
	GIC + Lyophilized miswak			3 weeks	9.05(1.31)	
	GIC + ZOE-AgNPs				11.55(1.78)	
	GIC + Chlorhexidine diacetate (CHX)				14.17(1.07)	
	GIC + ZOE-AgNPs + CHX				18.81(1.43)	
	Control (Conventional GIC)				0(1.31)	
Ashour et al. 2022 [16]	GIC (control)	*Staphylococcus aureus*		1 Day	NA	
	GIC + 1.5% metronidazole				14 (1.62)	
	99.5% GIC + 0.5% TVE-CuNPs				20 (1.53)	
	98% GIC + 0.5% TVE-CuNPs + 1.5% metronidazole				29 (1.11)	
	99.5% GIC + 0.5% AgNPs				24 (1.41)	
	98% GIC + 0.5% AgNPs + 1.5% metronidazole				30 (1.71)	
	GIC (control)			2 Weeks	NA	
	GIC + 1.5% metronidazole				13 (1.52)	
	99.5% GIC + 0.5% TVE-CuNPs				17 (1.21)	
	98% GIC + 0.5% TVE-CuNPs + 1.5% metronidazole				22 (1.27)	
	99.5% GIC + 0.5% AgNPs				19 (0.57)	
	98% GIC + 0.5% AgNPs + 1.5% metronidazole				23 (1.52)	
	GIC (control)			1 Month	NA	
	GIC + 1.5% metronidazole				11 (0.57)	
	99.5% GIC + 0.5% TVE-CuNPs				15 (1.35)	
	98% GIC + 0.5% TVE-CuNPs + 1.5% metronidazole				19 (1.56)	
	99.5% GIC + 0.5% AgNPs				16 (1)	
	98% GIC + 0.5% AgNPs + 1.5% metronidazole				18 (1.15)	
	GIC (control)	*S mutans*	Disc diffusion test	1 Day	NA	
	GIC + 1.5% metronidazole				18 (1.93)	
	99.5% GIC + 0.5% TVE-CuNPs				19 (1.26)	
	98% GIC + 0.5% TVE-CuNPs + 1.5% metronidazole				26 (1.34)	
	99.5% GIC + 0.5% AgNPs				20 (1.65)	
	98% GIC + 0.5% AgNPs + 1.5% metronidazole				27 (1.35)	
	GIC (control)			2 Weeks	NA	
	GIC + 1.5% metronidazole				16 (1.67)	
	99.5% GIC + 0.5% TVE-CuNPs				16 (1.31)	
	98% GIC + 0.5% TVE-CuNPs + 1.5% metronidazole				21 (1.88)	
	99.5% GIC + 0.5% AgNPs				18 (1.53)	
	98% GIC + 0.5% AgNPs + 1.5% metronidazole				23 (1.93)	
	GIC (control)			1 month	NA	
	GIC + 1.5% metronidazole				12 ± 1.58	
	99.5% GIC + 0.5% TVE-CuNPs				13 ± 1.55	
	98% GIC + 0.5% TVE-CuNPs + 1.5% metronidazole				18 ± 1.17	
	99.5% GIC + 0.5% AgNPs				14 ± 1.66	
	98% GIC + 0.5% AgNPs + 1.5% metronidazole				17 ± 1.69	
Ahalya et al. 2021 [13]	Glass Ionomer Cement	*Streptococcus mutans*	Agar Well Diffusion Method	24 h	0 mm	
	GIC + ZnO nanoparticles				Range from 10 to 15 mm	
Enan et al. 2021 [19]	conventional GIC	*S. mutans*	Kirby-Bauer method	24 h	8 (1.14)	
	GIC with AgNPs				18 (2.5)	
	GIC with amoxicillin				8 (0.7)	
	GIC with both AgNPs and amoxicillin				29 (1.14)	
	conventional GIC,			1 Week	0	
	GIC with AgNPs				13.8 (0.83)	
	GIC with amoxicillin				0	
	GIC with both AgNPs and amoxicillin				20 (1.14)	
	conventional GIC,			3Week	0	
	GIC with AgNPs				11 (0.84)	
	GIC with amoxicillin				0	
	GIC with both AgNPs and amoxicillin				15 (1.04)	
	conventional GIC,	*S. aureus*		24 h	9	
	GIC with AgNPs				20	
	GIC with amoxicillin				9	
	GIC with both AgNPs and amoxicillin				30	
	conventional GIC,			1 Week	0	
	GIC with AgNPs				14.8	
	GIC with amoxicillin				0	
	GIC with both AgNPs and amoxicillin				23	
	conventional GIC,			3Week	0	
	GIC with AgNPs				12	
	GIC with amoxicillin				0	
	GIC with both AgNPs and amoxicillin				16	
Siddiqui et al. 2024 [33]	GIC	*Streptococcus mutans*	growth inhibition zone	24 h	6	
	0.2 SIAgNPs-GIC				9.3	
	0.1 SIAgNPs-GIC				8.7	
	0.05 SIAgNPs-GIC				7.7	
	0.025 SIAgNPs-GIC				7.3	
	GIC	Lactobacillus acidophilus			10.5	
	0.2 SIAgNPs-GIC				12.5	
	0.1 SIAgNPs-GIC				11	
	0.05 SIAgNPs-GIC				10.45	
	0.025 SIAgNPs-GIC				10.4	
Saddique et al. 2025 [36]	CuNPs	*MRSA*	Antibacterial activity	24 h	13 ± 0.81	
		K. pneumonia			16 ± 1.10	
		P. aeruginosa			14 ± 1.37	
		E. faecalis			14 ± 0.39	
		MRSA	minimum inhibitory concentration		400 ± 2.54	
		K. pneumonia			400 ± 1.67	
		P. aeruginosa			500 ± 2.43	
		E. faecalis			400 ± 1.87	
Sundeep et al. 2017 [35]	2 µg/mL	Escherichia coli	Kirby–Bauer and Stokes methods	24 h	0.5	
	4 µg/mL				0.7	
	6 µg/mL				0.9	
	8 µg/mL				1.2	
	2µg/mL	Staphylococcus aureus			0.7	
	4 µg/mL				0.9	
	6 µg/mL				1.1	
	8 µg/mL				1.5	
Guo et al. 2025 [22]	Negative control	Biofilm (*S. mutans, S.sobrinus, L.fermentum and L.rhamnosu*s)	ratio (%) of live to total bacteria	–	98.55 + 0.1	
	0.05% AgNW-GIC				94.57 + 0.1	
	0.1% AgNW-GIC				87.68 + 2.54	
	0.3% AgNW-GIC				55.07 + 0.36	
	0.5% AgNW-GIC				4.71 + 2.54	
	0.5% AgNP				6.88 + 1.45	
Jain et al. 2024 [24]	control	*Streptococcus mutans*	minimal inhibitory concentration (MIC)	1 h	0.467 ± 0.00083	
				2 h	0.444 ± 0.00051	
				3 h	0.402 ± 0.00051	
				4 h	0.375 ± 0.00051	
				5 h	0.321 ± 0.00083	
	3% ZrNP			1 h	0.463 ± 0.00083	
				2 h	0.422 ± 0.00098	
				3 h	0.366 ± 0.00083	
				4 h	0.312 ± 0.00098	
				5 h	0.265 ± 0.00063	
	5% ZrNP			1 h	0.422 ± 0.00083	
				2 h	0.376 ± 0.00083	
				3 h	0.321 ± 0.00132	
				4 h	0.284 ± 0.00081	
				5 h	0.238 ± 0.00083	
	10% ZrNP			1 h	0.563 ± 0.00040	
				2 h	0.565 ± 0.00081	
				3 h	0.597 ± 0.00116	
				4 h	0.634 ± 0.00147	
				5 h	0.676 ± 0.00103	
	control	*Lactobacillus acidophilus*		1 h	0.507 ± 0.0006	
				2 h	0.510 ± 0.0008	
				3 h	0.512 ± 0.0010	
				4 h	0.474 ± 0.0016	
				5 h	0.444 ± 0.0013	
	3% ZrNP			1 h	0.477 ± 0.0012	
				2 h	0.488 ± 0.0005	
				3 h	0.443 ± 0.0006	
				4 h	0.401 ± 0.0008	
				5 h	0.366 ± 0.0008	
	5% ZrNP			1 h	0.421 ± 0.0008	
				2 h	0.415 ± 0.0016	
				3 h	0.402 ± 0.0009	
				4 h	0.355 ± 0.0008	
				5 h	0.311 ± 0.0012	
	10% ZrNP			1 h	0.612 ± 0.0010	
				2 h	0.638 ± 0.0012	
				3 h	0.658 ± 0.0008	
				4 h	0.697 ± 0.0008	
				5 h	0.721 ± 0.0008	
Jairam et al. 2024 [25]	8%CeO_2_-GIC	*Streptococcus mutans*	CFUs	72 h	1.60 ± 0.89	
	6%CeO_2_-GIC				15.40 ± 4.45	
	4% CeO_2_-GIC				168.00 ± 27.75	
	2% CeO_2_-GIC				5.20 ± 2.28	
	GIC				18.00 ± 3.94	
Mansoor et al. 2022 [26]	TiO_2_ nanoparticles	*Streptococcus mutans*	Cell viability	Control	100 ± 1.075	
				Day 1	98.64 ± 1.076	
				Day 3	96.07 ± 1.077	
				Day 7	93.19 ± 1.078	
				Day 21	91.71 ± 1.079	
				Day 30	90.13 ± 1.080	
Goldani Moghadam et al. 2022 [29]	GIC-AgNPs	*S. mutans*	growth inhibition zone	72 h	10.17 ± 1.04	
		*L. acidophilus*			11.67 ± 0.29	
		*L. casei*			8.16 ± 0.29	
		*E. faecalis*			No inhibition	
		*S. aureus*			8.67 ± 0.76	
	Ampicillin (10 µg/ml)	*S. mutans*			51.33 ± 1.04	
		*L. acidophilus*			28.88 ± 1.07	
		*L. casei*			26.83 ± 0.67	
		*E. faecalis*			32.17 ± 0.76	
		*S. aureus*			32.83 ± 0.29	

### Mechanical properties results

Mechanical properties were evaluated in 11 of the 25 included studies (44%). Compressive strength was the most frequently investigated mechanical outcome (11 studies), followed by microhardness (8 studies) and flexural strength (4 studies). Overall, most studies reported improvements in mechanical performance following incorporation of green-synthesized nanoparticles, although the magnitude of improvement varied according to nanoparticle type and concentration.

Formulations containing chitosan–titania–zirconia–hydroxyapatite frequently demonstrated marked increases in compressive strength compared to unmodified GIC, with several reporting enhancements exceeding 20–30%. Zirconia-based and hydroxyapatite-based nanoparticles similarly contributed to notable improvements in compressive strength and microhardness. In contrast, formulations incorporating biologically synthesized silver nanoparticles produced more variable outcomes; while some demonstrated modest improvements, others reported decreased microhardness and the development of surface irregularities or microcracks (Table 6).

**Table 6:** Mechanical properties results

Study	Groups	Property	Mean (SD)
Alex et al. 2025 [14]	GIC WITH 3% oF (Ch-Ti-Zr-HA) NPS	Flexural Strength (Mpa)	27.12 (0.045)
	GIC WITH 5% oF (Ch-Ti-Zr-HA) NPS		31.67 (0.247)
	GIC WITH 10% oF (Ch-Ti-Zr-HA) NPS		34.71 (0.223)
	GIC CONTROL		16.86 (0.019)
	GIC WITH 3% oF (Ch-Ti-Zr-HA) NPS	Compression strength (MPa)	178.57 (0.105)
	GIC WITH 5% oF (Ch-Ti-Zr-HA) NPS		189.75 (0.052)
	GIC WITH 10% oF (Ch-Ti-Zr-HA) NPS		197.29 (0.253)
	GIC CONTROL		167.85 (1.025)
	GIC WITH 3% oF (Ch-Ti-Zr-HA) NPS	Hardness	45.44 (0.286)
	GIC WITH 5% oF (Ch-Ti-Zr-HA) NPS		48.58 (0.144)
	GIC WITH 10% oF (Ch-Ti-Zr-HA) NPS		50.35 (0.232)
	GIC CONTROL		41.65 (0.144)
Ekrikaya et al. 2025 [18]	GIC (control group)	Hardness	101.13 (3)
	GIC + Green synthesized Ag NPs		98.26 (2.92)
	GIC + Green synthesized Ag@MoS2 nanocomposite		97.51 (2.7)
	GIC + Chemically synthesized Ag NPs		100.88 (1.69)
	GIC + Chemically synthesized Ag@MoS2		97.83 (2,39)
Ashour et al. 2022 [16]	GIC (control)	Compression strength (MPa)	41.2 (3.4 MPa)
	GIC + 1.5% metronidazole		42.8 (2.8 MPa)
	99.5% GIC + 0.5% TVE-CuNPs		44.2 (3.8 MPa)
	98% GIC + 0.5% TVE-CuNPs + 1.5% metronidazole		43.9 (3.7 MPa)
	99.5% GIC + 0.5% AgNPs		45.9 (4.1 MPa)
	98% GIC + 0.5% AgNPs + 1.5% metronidazole		45 (4.5 MPa)
Enan et al. 2021 [19]	conventional GIC,	Compression strength (MPa)	44.4 (3.7)
	GIC with AgNPs		45.6 (5.3)
	GIC with amoxicillin		38.4 (4.2)
	GIC with both AgNPs and amoxicillin		45.3 (6.3)
Qasim et al. 2021 [31]	GIC	Compression strength (MPa)	24.86 (1.77)
	SNP 10%		38.9 (8.03)
	SNP 30%		20.23 (5.72)
	SNP 50%		30.45 (2.93)
		Hardness	Microhardness 0 day
	GIC		25.21 (1.17)
	SNP 10%		33.43 (1.07)
	SNP 30%		43.47 (0.64)
	SNP 50%		38.99 (1.6)
			Microhardness 1 day
	GIC		53.51 (2.03)
	SNP 10%		52.23 (1.92)
	SNP 30%		65.47 (0.86)
	SNP 50%		71.24 (1.71)
			Microhardness 4 days
	GIC		71.24 (1.18)
	SNP 10%		69.32 (1.6)
	SNP 30%		63.55 (0.96)
	SNP 50%		67.4 (1.39)
			Microhardness 7 days
	GIC		73.7 (1.92)
	SNP 10%		76.48 (1.17)
	SNP 30%		78.61 (1.39)
	SNP 50%		78.5 (0.97)
Ravi et al. 2024 [32]	Group I (3%Ch-Ti-Zr-HA NPs)	Flexural Strength (Mpa)	28.1 (2.32)
	Group II (5%Ch-Ti-Zr-HA NPs)		30.96 (1.65)
	Group III (10%Ch-Ti-Zr-HA NPs)		32.67 (4.76)
	Group IV (GIC)		16.86 (0.19)
	Group I (3%Ch-Ti-Zr-HA NPs)	Compression strength (MPa)	178.57 (0.1)
	Group II (5%Ch-Ti-Zr-HA NPs)		188.91 (2.9)
	Group III (10%Ch-Ti-Zr-HA NPs)		196.45 (2.83)
	Group IV (GIC)		167.85 (1.02)
	Group I (3%Ch-Ti-Zr-HA NPs)	Hardness	45.54 (1.84)
	Group II (5%Ch-Ti-Zr-HA NPs)		48.54 (0.86)
	Group III (10%Ch-Ti-Zr-HA NPs)		50.29 (1.35)
	Group IV (GIC)		41.61 (0.94)
Guo et al. 2025 [22]	Conventional GIC	Compression strength (MPa)	45.83 (3.13)
	0.05% AgNW-GIC		40 (5.76)
	0.1% AgNW-GIC		40 (4.05)
	0.3% AgNW-GIC		42.67 (2.34)
	0.5% AgNW-GIC		41.67 (1.51)
	0.5% AgNP-GIC		44.33 (2.42)
	Conventional GIC	Hardness	541.2 (5.8)
	0.05% AgNW-GIC		529.5 (6.6)
	0.1% AgNW-GIC		492.6 (9.7)
	0.3% AgNW-GIC		487 (1.2)
	0.5% AgNW-GIC		470.9 (5.3)
	0.5% AgNP-GIC		457.8 (± 2.7)
Ilancheran et al. 2024 [23]	group I nHA 3%	Compression strength (MPa)	172.55 (0.769)
	group ii nHA 5%		178.16 (0.76)
	group iii nHA 10%		182.45 (0.95)
	group iv contorl 0%		162.46 (1.606)
Jain et al. 2024 [24]	Group I GIC + 3% zirconia nanoparticles	Compression strength (MPa)	180.48 (1.024)
	Group II GIC + 5% zirconia nanoparticles		191.25 (0.524)
	Group IIIGIC + 10% zirconia nanoparticles		197.52 (0.755)
	Group IV Control (conventional GIC)		167.22 (1.235)
Jairam et al. 2024 [25]	Group A 8% CeO₂-GIC	Compression strength (MPa)	244.9 (3.4)
	Group B 6% CeO₂-GIC		212.1 (2.8)
	Group C 4% CeO₂-GIC		207.6 (5.3)
	Group D 2% CeO₂-GIC		200.4 (1.3)
	Group E (Control)		177.2 (1.2)
	Group A 8% CeO₂-GIC	Hardness	40.90 (1.54)
	Group B 6% CeO₂-GIC		39.22 (0.94)
	Group C 4% CeO₂-GIC		37.2 (1.32)
	Group D 2% CeO₂-GIC		30.52 (0.97)
	Group E (Control)		30.56 (1.39)
	Group A 8% CeO₂-GIC	Diametral tensile Strength (MPa)	33.46 (1.25)
	Group B 6% CeO₂-GIC		30.29 (0.91)
	Group C 4% CeO₂-GIC		28.29 (0.54)
	Group D 2% CeO₂-GIC		25.78 (1.17)
	Group E (Control)		20.68 (0.54)
Mansoor et al. 2022 [26]	control group = 1 (0 wt% TiO_2_-GIC),	Flexural Strength (Mpa)	16.11 (0.443)
	2 (3 wt% TiO_2_-GIC		21.41 (0.444)
	3 (5 wt% TiO_2_-GIC)		26.39 (0.43)
	4 (7 wt% TiO_2_-GI		19.60 (0.446)
	5 (10 wt% TiO-GIC)		17.11 (0.24)
	control group = 1 (0 wt% TiO_2_-GIC),	Compression strength (MPa)	7.63 (0.6)
	2 (3 wt% TiO_2_-GIC		11.39 (0.6)
	3 (5 wt% TiO_2_-GIC)		15.51 (0.39)
	4 (7 wt% TiO_2_-GI		11.9 (0.6)
	5 (10 wt% TiO-GIC)		8.10 (0.37)
Mansoor et al. 2025 [27]	Conventional GIC (0%)	Shear strength (Mpa)	2.17 (0.2)
	Smart Titania-GIC (3%)		2.99 (0.45)
	Smart Titania-GIC (5%)		4.93 (0.74)
	Smart Titania-GIC (7%)		3.96 (0.3)
	Smart Titania-GIC (10%)		3.44 (0.57)
Palani et al. 2025 [30]	Group I (3%Ch-Ti-Zr-HA NPs)	Roughness	1.46 (0.88)
	Group II (5%Ch-Ti-Zr-HA NPs)		3.42 (1.71)
	Group III (10%Ch-Ti-Zr-HA NPs)		3.92 (2.42)
	Group IV (GIC)		2.13 (1.06)
Ganesh et al. 2024 [21]	Gloss test prebrushing	Roughness	
	3% Ch–Ti–Zr–HA		39.91 ± 1.55
	5% Ch–Ti–Zr–HA		40.87 ± 0.86
	10% Ch–Ti–Zr–HA		28.87 ± 1.03
	control		40.00 ± 2.16
	Gloss test postbrushing		
	3% Ch–Ti–Zr–HA		40.72 ± 0.87
	5% Ch–Ti–Zr–HA		42.85 ± 3.21
	10% Ch–Ti–Zr–HA		32.75 ± 2.03
	control		40.28 ± 0.81
Guo et al. 2025 [22]	Conventional GIC	Roughness	18.104
	0.05% AgNW-GIC		19.724
	0.1% AgNW-GIC		18.036
	0.3% AgNW-GIC		22.663
	0.5% AgNW-GIC		16.782
	0.5% AgNP-GIC		26.006

Flexural strength was less frequently assessed, but the available studies consistently demonstrated improved performance in GICs modified with hybrid or multi-phase nanoparticles. Notably, chitosan-based nanoparticle blends yielded the highest flexural strength values, typically doubling those of the corresponding control GICs. These findings suggest that nanoparticles with organic–inorganic hybrid structures may provide more effective enhancement of flexural resistance.

### Fluoride release results

Fluoride release was investigated in two studies, each demonstrating that nanoparticle incorporation did not diminish the characteristic fluoride-releasing behavior of GIC. In some cases, such as those employing hydroxyapatite or chitosan–metal hybrids, fluoride release was modestly enhanced over extended periods, likely due to alterations in matrix porosity and ion-exchange dynamics (Table 7).

**Table 7:** Fluoride release

Study	Groups	Time Point	Mean (SD)
Guo et al. 2025 [22]	Conventional GIC	1 st Day	3.177 ± 0.193
	0.05% AgNW-GIC		3.865 c 0.465
	0.1% AgNW-GIC		4.429 ± 2.061
	0.3% AgNW-GIC		4.99
	0.5% AgNW-GIC		3.865 c 0.465
	0.5% AgNP-GIC		5.4
	Conventional GIC	3rd Day	3.325 ± 1.227
	0.05% AgNW-GIC		5.58 ± 0.98
	0.1% AgNW-GIC		5.43 ± 1.13
	0.3% AgNW-GIC		4.55
	0.5% AgNW-GIC		5.58 ± 0.98
	0.5% AgNP-GIC		7.1
	Conventional GIC	7th Day	4.478 ± 0.534
	0.05% AgNW-GIC		5.7 ± 0.61
	0.1% AgNW-GIC		6.319 ± 1.2
	0.3% AgNW-GIC		6.466
	0.5% AgNW-GIC		5.7 ± 0.61
	0.5% AgNP-GIC		6.66
	Conventional GIC	14th Day	5.11 ± 1.025
	0.05% AgNW-GIC		5.38 ± 0.4175
	0.1% AgNW-GIC		5.82 ± 0.646
	0.3% AgNW-GIC		4.5
	0.5% AgNW-GIC		5.38 ± 0.4175
	0.5% AgNP-GIC		5.53
Dhivyasri et al. 2025 [17]	3% OF (Ch-Ti-Zr-HA) NPS	Day 1	129.04 ± 0.146
	5% OF (Ch-Ti-Zr-HA) NPS		153.96 ± 1.015
	10% OF (Ch-Ti-Zr-HA) NPS		170.03 ± 0.512
	GIC Control		117.11 ± 0.098
	3% OF (Ch-Ti-Zr-HA) NPS	Day 3	94.28 ± 0.194
	5% OF (Ch-Ti-Zr-HA) NPS		126.10 ± 0.109
	10% OF (Ch-Ti-Zr-HA) NPS		164.83 ± 0.427
	GIC Control		48.13 ± 0.073
	3% OF (Ch-Ti-Zr-HA) NPS	Day 7	78.16 ± 0.149
	5% OF (Ch-Ti-Zr-HA) NPS		98.430 ± 0.408
	10% OF (Ch-Ti-Zr-HA) NPS		155.10 ± 0.275
	GIC Control		40.18 ± 0.164
	3% OF (Ch-Ti-Zr-HA) NPS	Day 14	68.15 ± 0.151
	5% OF (Ch-Ti-Zr-HA) NPS		70.36 ± 0.512
	10% OF (Ch-Ti-Zr-HA) NPS		126.20 ± 0.309
	GIC Control		31.15 ± 0.204
	3% OF (Ch-Ti-Zr-HA) NPS	Day 28	44.18 ± 0.183
	5% OF (Ch-Ti-Zr-HA) NPS		54.75 ± 1.172
	10% OF (Ch-Ti-Zr-HA) NPS		76.16 ± 0.107
	GIC Control		24.05 ± 0.151

### Color stability and other properties results

Color stability was evaluated in four studies, where low nanoparticle concentrations maintained clinically acceptable ΔE values, though higher loadings occasionally produced noticeable discoloration (Supplementary File).

## Discussion

Across the reviewed literature, nanoparticle modification of glass ionomer cement (GIC) consistently improved both mechanical performance and antimicrobial behavior, although the underlying mechanisms varied according to particle type and concentration. The reviewed studies collectively highlight that the incorporation of nanoparticles into GICs enhances antimicrobial activity through a variety of mechanisms, often centered on bacterial membrane disruption, oxidative stress generation, and interference with essential cellular processes. Although the general outcome across studies is consistent, improved antibacterial performance, the specific explanations vary depending on the type and behavior of the nanoparticles used.

Overall, the available evidence consistently supports the beneficial effect of green-synthesized nanoparticles on the antibacterial performance and mechanical properties of glass ionomer cement. Despite variability in nanoparticle composition, synthesis protocols, and testing methodologies, most studies demonstrated favorable outcomes, suggesting that the observed improvements are robust across different experimental conditions. Nevertheless, methodological heterogeneity precluded quantitative pooling of the data, and the conclusions should therefore be interpreted within the context of a qualitative evidence synthesis.

Across multiple studies, the antibacterial activity of green-synthesized nanoparticles was consistently attributed to reactive oxygen species (ROS) generation, disruption of bacterial cell membranes, and interference with intracellular metabolic pathways. These mechanisms were reported irrespective of nanoparticle composition, although calcium-containing nanoparticles additionally contributed through ion-mediated effects, whereas silver and copper nanoparticles exerted their antibacterial action predominantly through metal-ion release and protein interaction [13, 14, 23, 25].

Beyond ROS generation, the antibacterial activity of green-synthesized nanoparticles is mediated through several complementary mechanisms. First, nanoparticles directly disrupt bacterial cell membranes by interacting with membrane phospholipids, increasing membrane permeability and ultimately causing leakage of intracellular contents. Second, the sustained release of metal ions, particularly silver and copper ions, interferes with bacterial proteins by binding to sulfhydryl groups, inactivating essential enzymes and disrupting metabolic pathways. Third, nanoparticles penetrate bacterial cells and interact with nucleic acids, impairing DNA replication, transcription, and protein synthesis. In addition, electrostatic attraction between positively charged nanoparticles and negatively charged bacterial surfaces facilitates close contact, enhancing membrane damage and cellular uptake. Finally, several studies demonstrated inhibition of extracellular polysaccharide (EPS) production and biofilm formation, thereby reducing bacterial adhesion, colonization, and virulence. Collectively, these complementary mechanisms explain the broad-spectrum antibacterial activity consistently observed in green-synthesized nanoparticle-modified glass ionomer cements [16, 18–20, 22, 35].

Metal-based nanoparticles, particularly silver and copper, further enhanced antibacterial activity through sustained metal-ion release, protein denaturation, and interference with bacterial nucleic acids. Several investigations further demonstrated synergistic antibacterial effects when nanoparticles were combined with conventional antimicrobial agents, suggesting that nanoparticle incorporation may potentiate existing antibacterial strategies [16, 18, 33].

Across multiple studies, improvements in compressive strength and flexural resistance were attributed to the micro-filler effect of nanoparticles, which reduced internal porosity, increased packing density, promoted stronger particle–matrix interactions, and enhanced cross-linking within the cement matrix [14, 23, 24, 26, 27].

Comparable findings were reported, as reduced porosity was observed when zirconia displaced air pockets during mixing [24], and improved microhardness and wear resistance were confirmed in zirconia-reinforced GICs [24]. Optimal nanoparticle concentration was consistently emphasized, with mechanical stability and abrasion resistance shown to be enhanced at 5% incorporation, whereas higher loading was found to compromise gloss and surface quality [30].

However, not all nanoparticles were found to provide mechanical benefits. It was observed that silver nanoparticles dispersed in the liquid phase of GIC failed to chemically integrate with the cement matrix, resulting in no significant mechanical improvement and potentially introducing pores that act as stress concentrators [31]. Increased surface cracking and darker discoloration were similarly reported following nanosilver addition, corresponding to reduced microhardness [22]. Collectively, these findings indicate that mechanical reinforcement is governed not only by particle size and distribution but also by chemical compatibility and controlled concentration.

Collectively, these findings demonstrate that the enhanced clinical performance of nanoparticle-modified glass ionomer cements results from the combined contribution of improved matrix integrity and broad-spectrum antimicrobial activity. The synergistic interaction between mechanical reinforcement and multiple antibacterial mechanisms supports the potential of green-synthesized nanoparticles as multifunctional modifiers for restorative dental materials.

The available evidence indicates that nanoparticle-reinforced GICs benefit from structural strengthening mechanisms, such as void filling, increased cross-linking, and enhanced density, as well as antimicrobial pathways including ROS generation, metal-ion release, electrostatic interactions, and biofilm inhibition. Nevertheless, the importance of optimal nanoparticle concentration and chemical compatibility is repeatedly emphasized, as inappropriate incorporation may fail to improve or may even compromise material properties. The current literature therefore supports nanoparticle integration as an effective strategy to develop GIC materials with superior mechanical resilience and antimicrobial functionality.

An additional consideration when interpreting the findings of this review is the variability among the commercial glass ionomer cements used in the included studies. Different GIC formulations vary in their glass particle composition and size, polyacid chemistry, powder-to-liquid ratio, setting characteristics, and fluoride content. These formulation-dependent differences may influence nanoparticle dispersion, interfacial interactions with the cement matrix, mechanical reinforcement, fluoride release, and antibacterial performance. Consequently, the beneficial effects observed for a particular nanoparticle formulation in one commercial GIC cannot be assumed to be directly applicable to other GIC systems. Future investigations should therefore evaluate green-synthesized nanoparticles using standardized testing protocols across multiple commercially available GIC formulations to determine the consistency and generalizability of their effects.

### Comparison of green-synthesized and chemically synthesized nanoparticles

Building upon the diverse antimicrobial mechanisms described in the literature, it is also essential to consider how the method of nanoparticle synthesis influences their behavior within GICs and their resulting antibacterial performance. Nanoparticles can be produced using chemical, physical, or biological (green) methods, and this choice of synthesis pathway shapes not only their physicochemical characteristics but also their biocompatibility and clinical relevance [3, 4].

In recent years, green synthesis has gained growing attention for dental applications due to its reliance on natural reducing agents, such as plant extracts, enzymes, and proteins, that eliminate the toxic solvents and harsh reducing chemicals typically required in conventional chemical synthesis. Unlike conventional chemical routes, which often require hazardous reducing agents and stabilizers and may leave residual chemicals unsuitable for biomedical applications, green synthesis offers a non-toxic, biocompatible, environmentally friendly, and cost-effective alternative. Plant extracts are particularly favored because they are abundant, cheap, and naturally rich in biomolecules capable of reducing metal ions while simultaneously stabilizing the resulting nanoparticles. Green tea extract, for instance, contains epigallocatechin-3-gallate (EGCG), an efficient reducing and capping agent capable of reducing Ag⁺ to Ag⁰ while preventing nanoparticle aggregation. Silver nanoparticles produced through green tea extract demonstrate broad-spectrum antimicrobial activity against organisms such as *Streptococcus mutans*, *Staphylococcus aureus*, *Pseudomonas aeruginosa*, *Klebsiella pneumoniae*, *Salmonella enterica*, and *Escherichia coli* [38].

In contrast, chemically synthesized nanoparticles often exhibit highly controlled size, uniformity, and stability; however, the use of toxic precursors and stabilizers poses limitations for biomedical applications. Their synthesis may yield nanoparticles with strong antibacterial effects but introduces safety concerns, cytotoxic risk, and environmental burden, issues not associated with green synthesis. These contrasting features have driven many researchers toward exploring biological routes, especially for dental materials intended for direct tissue contact [3, 4].

These findings highlight an important point: although green synthesis is inherently safer and more environmentally friendly, it does not invariably produce nanoparticles with superior antibacterial activity and presents several challenges related to reproducibility and large-scale production. Several factors may explain this. Plant-derived phytochemicals can coat the nanoparticle surface, hindering the direct contact needed for maximum antimicrobial potency or slowing silver ion release. Additionally, green-synthesized nanoparticles may exhibit lower colloidal stability, making them prone to aggregation, which reduces active surface area and antimicrobial efficiency. Variability in plant extract composition can also cause inconsistency in particle size and properties [3, 4].

Despite these advantages, several important limitations of green nanoparticle synthesis should be acknowledged. The physicochemical characteristics of green-synthesized nanoparticles may vary considerably between batches because the composition of plant extracts is influenced by plant species, geographical origin, cultivation conditions, harvesting season, extraction procedures, and storage conditions [3, 4]. Such variability can affect nanoparticle size, morphology, surface chemistry, and biological activity, thereby reducing reproducibility across studies [3, 4]. Furthermore, the absence of standardized synthesis protocols presents challenges for quality control and large-scale industrial production. Achieving consistent nanoparticle characteristics under Good Manufacturing Practice (GMP) conditions remains difficult, potentially limiting clinical translation and commercial application. Future research should therefore prioritize standardized synthesis methods, comprehensive physicochemical characterization, and scalable manufacturing strategies to facilitate the successful implementation of green-synthesized nanoparticles in restorative dental materials [2–4].

The findings of the present systematic review are generally consistent with those of previously published reviews investigating nanoparticle-modified glass ionomer cements. Recent reviews have similarly concluded that nanoparticle incorporation enhances the antibacterial activity and mechanical performance of GICs while emphasizing the importance of nanoparticle concentration, dispersion, and compatibility with the cement matrix [1, 2]. However, unlike previous reviews, which broadly evaluated nanomaterials or included both chemically synthesized and green-synthesized nanoparticles, the present review exclusively synthesized evidence related to green-synthesized nanoparticles. This focused approach enabled a comprehensive evaluation of their unique advantages, including environmentally sustainable synthesis, improved biocompatibility, and the contribution of phytochemicals to antimicrobial activity. Furthermore, the present review incorporated a formal methodological quality assessment using study design-specific risk-of-bias tools and systematically synthesized evidence across antimicrobial, mechanical, fluoride-release, color stability, and physicochemical outcomes, thereby providing a more focused and clinically relevant evaluation of this emerging field.

### Limitations and future directions

Despite the promising antimicrobial enhancements afforded by nanoparticle incorporation into glass ionomer cements (GIC), several limitations remain. First, nanoparticle distribution within the GIC matrix is often heterogeneous, leading to inconsistent mechanical and antimicrobial properties. Achieving uniform dispersion without agglomeration remains a significant technical challenge. Additionally, many studies report improved in vitro antimicrobial activity, but there is a lack of standardized in vivo and clinical data confirming long-term effectiveness and safety of these nanoparticle-modified materials.

The potential cytotoxicity and biocompatibility of nanoparticles are also concerns as the release of nanoparticles or ions over time could affect surrounding tissues, yet comprehensive toxicological evaluations are scarce. Moreover, the optimal nanoparticle concentration balancing antimicrobial efficacy without compromising mechanical strength and esthetics needs further elucidation. The variability in nanoparticle synthesis methods, particularly with emerging green synthesis routes, necessitates standardization of protocols to ensure reproducibility and consistent material performance.

Future research should focus on optimizing nanoparticle incorporation techniques to ensure uniform distribution, comprehensive in vivo and clinical trials to validate long-term safety and effectiveness, and detailed toxicological assessments to address biocompatibility concerns. Advances in green synthesis must be pursued alongside rigorous material characterization to capitalize on their biocompatible and sustainable advantages. Furthermore, exploring multifunctional nanoparticles that simultaneously enhance mechanical, antimicrobial, and remineralization properties holds great potential. Regulatory frameworks and standardized testing guidelines tailored for nanoparticle-modified GICs are essential to facilitate safe clinical adoption.

## Conclusions

Incorporation of green nanoparticles into glass ionomer cement significantly enhances its antimicrobial properties, offering promising potential to reduce microbial colonization and secondary caries in dental restorations. Nanoparticles such as zinc oxide, silver, copper, hydroxyapatite, and zirconia exhibit multifaceted antimicrobial mechanisms including bacterial membrane disruption, reactive oxygen species induction, and inhibition of biofilm formation. Green synthesis of nanoparticles provides an eco-friendly, biocompatible alternative to conventional chemical methods, leveraging natural biological compounds to produce stable, effective antimicrobial agents with reduced cytotoxicity risks.

## Supplementary Information

Supplementary Material 1.

## Data Availability

All data supporting the findings of this study are included within the manuscript and Supplementary file.
