# Supplementary material for: Green-synthesized nanoparticles for glass ionomer cement reinforcement: a systematic review of their antimicrobial, mechanical, and physicochemical properties
Source: BMC Oral Health. 2026 Oct 3;26:1920. doi: 10.1186/s12903-026-09657-w (PMC13637097; doi:10.1186/s12903-026-09657-w)
Supplement: Supplementary file 1 [file 12903_2026_9657_MOESM1_ESM.docx]

**Table S1**: shows color stability results

| Study | Group | Time point | ΔE |
| --- | --- | --- | --- |
| Ekrikaya et al. 2025[(18)](https://sciwheel.com/work/citation?ids=18290560&pre=&suf=&sa=0&dbf=0) | Control  C - Ag@MoS | After incorporation | NA |
|  | G- AgNP |  | 1.23±0.24 |
|  | G - Ag@MoS2 |  | 1.58±0.78 |
|  | C - AgNP |  | 1.38±0.56 |
|  | C - Ag@MoS2 |  | 1.17±0.69 |
| Paulraj et al. 2025 [(30)](https://sciwheel.com/work/citation?ids=18372259&pre=&suf=&sa=0&dbf=0) | Group I (3%Ch-Ti-Zr-HA NPs) | After incorporation | 0.646 ± 0.024 |
|  | Group II (5%Ch-Ti-Zr-HA NPs) |  | 0.499 ± 0.454 |
|  | Group III (10%Ch-Ti-Zr-HA NPs) |  | 1.672 ± 0.119 |
|  | Group IV (GIC) |  | 0.666 ± 0.027 |
| Guo et al. 2025 [(22)](https://sciwheel.com/work/citation?ids=18292132&pre=&suf=&sa=0&dbf=0) | 0.05% AgNW-GIC | After incorporation | 1.48+1.27 |
|  | 0.1% AgNW-GIC |  | 4.01+1.69 |
|  | 0.3% AgNW-GIC |  | 7.82+1.9 |
|  | 0.5% AgNW-GIC |  | 12.04+1.69 |
|  | 0.5% AgNP |  | 54.93+1.06 |
| Qasim et al. 2021 [(31)](https://sciwheel.com/work/citation?ids=18372253&pre=&suf=&sa=0&dbf=0) | GIC | 1h | 1.33+0.21 |
|  | SNP 10% |  | 4.03+0.07 |
|  | SNP 30% |  | 17.82+0.77 |
|  | SNP 50% |  | 30.47+1.54 |
|  | GIC | 24 h | 2.25+0.81 |
|  | SNP 10% |  | 6.86+0.51 |
|  | SNP 30% |  | 24.2+2.86 |
|  | SNP 50% |  | 33.2+0.26 |
|  | GIC | 4 days | 4.77+0.22 |
|  | SNP 10% |  | 6.16+1.38 |
|  | SNP 30% |  | 12.05+0.55 |
|  | SNP 50% |  | 30.01+1.86 |
|  | GIC | 7 days | 3.46+0.95 |
|  | SNP 10% |  | 17.41+0.79 |
|  | SNP 30% |  | 15.26+0.53 |
|  | SNP 50% |  | 34.3+0.6 |
|  | GIC | 14 days | 3.31+0.05 |
|  | SNP 10% |  | 6.5+0.74 |
|  | SNP 30% |  | 19.69+0.59 |
|  | SNP 50% |  | 29.24+1.67 |

**Table S2**: shows water sorption and solubility results

| Study | Group | water sorption and solubility |
| --- | --- | --- |
| Aradya et al. 2025 [(28)](https://sciwheel.com/work/citation?ids=18372250&pre=&suf=&sa=0&dbf=0) | control | 81.228 + 1.825 |
|  | 6% HAp | 79.012 + 1.598 |
|  | 8% HAp | 76.505 + 1.213 |
|  | 10% HAp | 74.501 + 2.003 |
| Wear Resistance | | |
| Sitaram et al. 2024 [(34)](https://sciwheel.com/work/citation?ids=18289008&pre=&suf=&sa=0&dbf=0) | 3%Ch-Ti-Zr-HA NPs | 0.57 + 0.056 |
|  | 5%Ch-Ti-Zr-HA NPs | 0.263 + 0.049 |
|  | 10%Ch-Ti-Zr-HA NPs | 0.399 + 0.059 |
|  | GIC | 0.581 + 0.072 |

**Table S3**. SYRCLE Risk of Bias assessment of the included in vivo animal study (Palani et al., 2025).

| **SYRCLE Domain** | **Judgment** | **Justification** |
| --- | --- | --- |
| **1. Sequence generation (selection bias)** | **Unclear risk** | The article states that rats were divided into conventional GIC and nano-modified GIC groups but does not describe how allocation was generated. |
| **2. Baseline characteristics (selection bias)** | **Low risk** | Experimental groups consisted of healthy Wistar albino rats receiving different materials under identical conditions. No important baseline imbalance was reported. |
| **3. Allocation concealment (selection bias)** | **Unclear risk** | No information was provided regarding concealment of allocation before implantation. |
| **4. Random housing (performance bias)** | **Unclear risk** | The manuscript does not indicate whether cages or housing positions were randomized. |
| **5. Blinding of caregivers/investigators (performance bias)** | **High risk** | No statement indicates that investigators performing the implantation or animal care were blinded to treatment allocation. |
| **6. Random outcome assessment (detection bias)** | **Unclear risk** | The order in which biochemical or histological assessments were performed was not reported to be randomized. |
| **7. Blinding of outcome assessor (detection bias)** | **High risk** | No indication that the histopathologist or laboratory personnel were blinded to group allocation. |
| **8. Incomplete outcome data (attrition bias)** | **Low risk** | All animals appear to have been accounted for at the predetermined evaluation periods, and no unexplained losses were reported. |
| **9. Selective outcome reporting (reporting bias)** | **Low risk** | All stated biochemical and histopathological outcomes were presented in the Results section. |
| **10. Other sources of bias** | **Low risk** | No additional obvious source of bias (e.g., inappropriate statistical analysis or major protocol deviations) was identified. Independent t-tests were used appropriately for the two-group comparison. |
